# Case Report: A case of giant left ventricular aneurysm resulting in LAD compression and stenosis

**DOI:** 10.3389/fcvm.2026.1754121

**Published:** 2026-03-18

**Authors:** Qun Wang, Longyu Li, Wenhua Lin

**Affiliations:** TEDA International Cardiovascular Hospital, Tianjin, China

**Keywords:** coronary compression, external compression, left ventricular aneurysm, multimodality imaging, myocardial infarction

## Abstract

We present the case of a 67-year-old male smoker who developed progressive heart failure despite successful primary PCI for an acute extensive anterior ST-elevation myocardial infarction. Initial echocardiography revealed an apical left ventricular aneurysm (LVA). Readmission 40 days later showed dramatic ventricular dilation, severely reduced LVEF (19%), and a large inferoapical aneurysm. Multimodality imaging was pivotal: repeat angiography demonstrated a new dynamic mid-LAD stenosis; IVUS confirmed extrinsic luminal compression without plaque; CMR and nuclear scintigraphy quantified extensive scar (69% of LV). This comprehensive workup excluded conventional causes and established a diagnosis of external mechanical compression of the mid-LAD by the expanding giant LVA, creating a vicious cycle of ischemia and remodeling. This case underscores that an LVA is not merely a passive scar but can be a dynamically disruptive entity. It highlights the critical role of advanced imaging in diagnosing this rare complication and illustrates the therapeutic dilemma when definitive surgery is declined.

## Introduction

## Case report

A 67-year-old man presented with a five-day history of intermittent chest pain. He had a significant smoking history (40 pack-years) and denied hypertension or diabetes. Physical examination revealed bilateral basal lung crackles. Cardiac auscultation indicated a regular rhythm without murmurs, and there was no peripheral edema. Admission electrocardiography showed sinus rhythm with pathological Q waves and ST-segment elevation in leads V1–V6. Laboratory findings included elevated BNP (1,840 pg/mL), CK-MB (10.6 ng/mL), myoglobin (150 ng/mL), troponin I (17.5 ng/mL), and D-dimer (1,070 ng/mL). Echocardiography revealed left ventricular dilation (LVEDd 53 mm), reduced systolic function (LVEF 42%), hypokinesis of the interventricular septum and left ventricular free wall, an apical left ventricular aneurysm (LVA), and diastolic dysfunction. A diagnosis of acute extensive anterior myocardial infarction was established.

**Figure 1:**
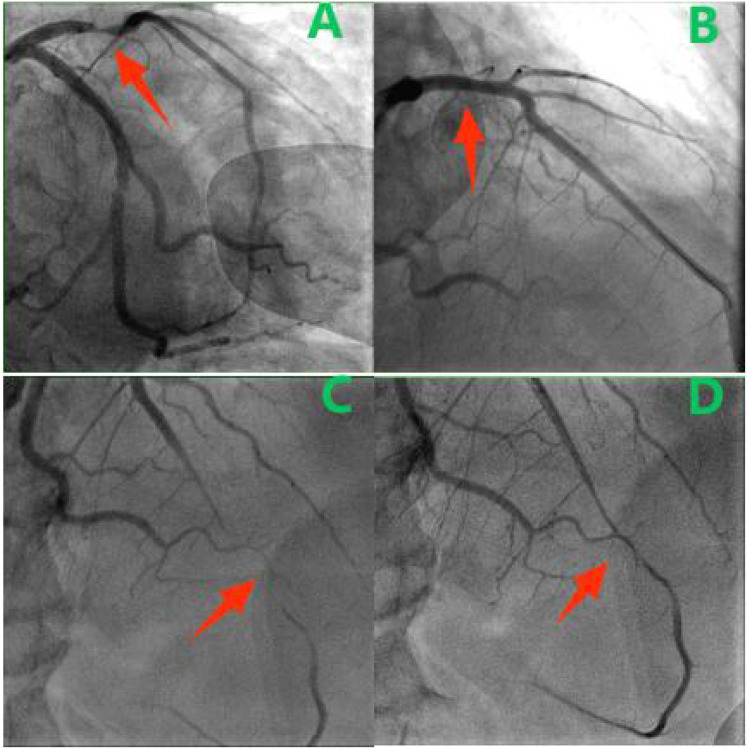
**(A)** Initial CAG showing stenosis (arrow) in the proximal LAD. **(B)** After stent placement. **(C,D)** Diastolic **(C)** and systolic **(D)** views showing dynamic mid-LAD compression (arrow).

The patient received antiplatelet therapy and guideline-directed medical therapy for heart failure. On hospital day 5, coronary angiography demonstrated a critical 95% segmental stenosis in the proximal left anterior descending artery (LAD) (Figure 1A), a 70% focal stenosis in the mid left circumflex artery, and patent left main and right coronary arteries. A 4.0 × 23 mm drug-eluting stent was successfully deployed in the proximal LAD (Figure 1B). Post-procedurally, the patient was angina-free. Follow-up echocardiography before discharge showed LVEDd 57 mm and LVEF 43%. Discharge medications included aspirin 100 mg daily, ticagrelor 90 mg twice daily, atorvastatin 20 mg daily, ramipril 1.25 mg daily, spironolactone 20 mg daily, and metoprolol tartrate 12.5 mg twice daily.

**Figure 2:**
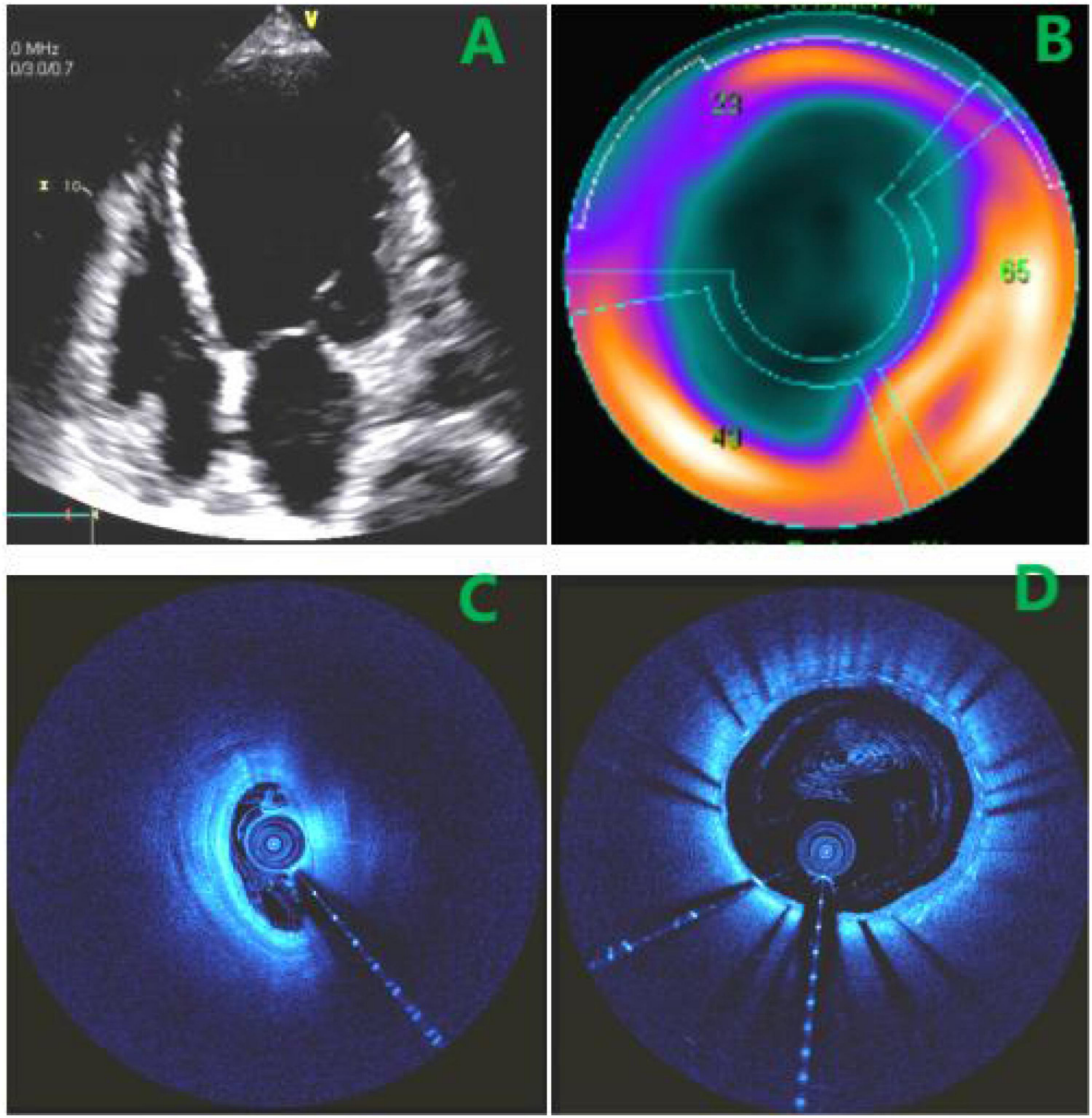
**(A)** large LV aneurysm on echo. **(B)** Perfusion scan:extensive scar (blue), minimal viable myocardium (red). **(C)** IVUS showing compressed mid-LAD lumen. **(D)** IVUS demonstrated patency of the stent in the proximal LAD.

Approximately 40 days after discharge, the patient was readmitted with progressive heart failure symptoms (dyspnea on ordinary exertion). Physical examination revealed basal lung crackles and an enlarged cardiac dullness border, without peripheral edema. Electrocardiography was unchanged. BNP had increased to 2,524 pg/mL. Echocardiography showed marked left ventricular dilation (LVEDd 62 mm), severely reduced LVEF (19%), a large mid-to-inferior left ventricular wall aneurysm, and moderate mitral regurgitation (Figure 2A). Repeat coronary angiography revealed a patent proximal LAD stent but a new diffuse 95% stenosis in the mid-LAD segment (Figures 1C,D). The stenosis exhibited dynamic phasic variation, unresponsive to intracoronary nitroglycerin. Myocardial bridging was excluded by review of angiograms. Intravascular ultrasound (IVUS) confirmed stent patency and showed a cyclically compressed, slit-like lumen in the mid-LAD segment without significant plaque (Figures 2C,D).

**Figure 3:**
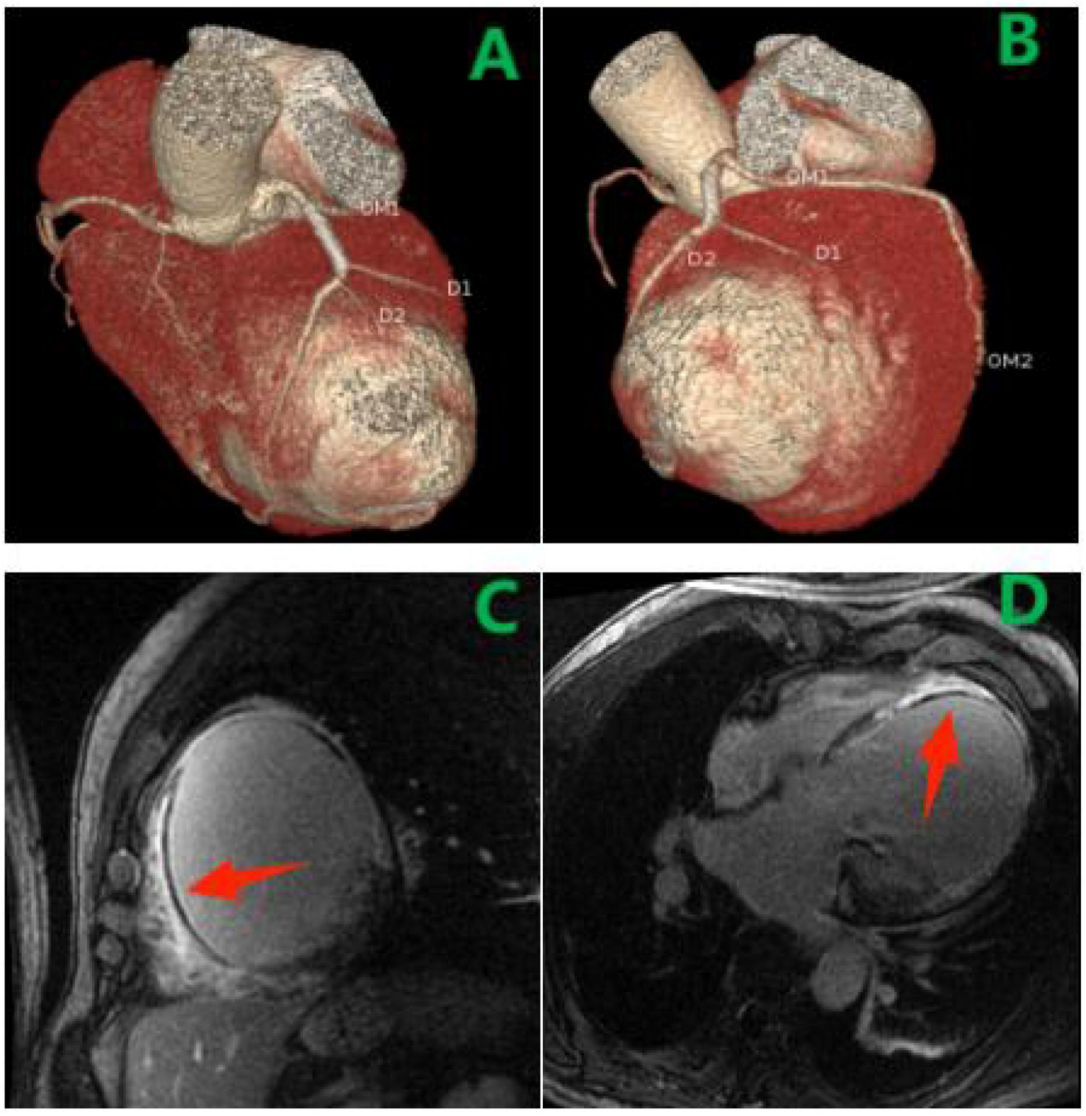
**(A,B)** CTA: giant aneurysm adjacent to LAD. **(C,D)** CMR LGE confirmed microvascular obstruction (MVO) within the apical septal subendocardial infarct (arrow).

Coronary computed tomography angiography (CTA) confirmed a giant left ventricular aneurysm and delineated its spatial relationship to the LAD (Figures 3A,B). Cardiac magnetic resonance imaging, including cine imaging (FIESTA sequence) for function and late gadolinium enhancement (PSIR sequence) for tissue characterization, revealed extensive fibrosis and microvascular obstruction in the anterior, lateral, septal, and apical segments of the left ventricle (Figures 3C,D). Myocardial perfusion scintigraphy revealed a scar involving approximately 69% of the left ventricular area (Figure 2B).

The cause of the new mid-LAD stenosis was systematically evaluated. Significant atherosclerosis progression was considered unlikely given the minimal plaque burden on IVUS. In-stent restenosis was excluded by IVUS confirmation of proximal stent patency. Coronary artery spasm was deemed improbable due to the lack of response to intracoronary nitroglycerin, and myocardial bridging was ruled out by careful angiographic review. Ultimately, the convergence of findings—dynamic phasic narrowing on angiography, extrinsic compression on IVUS, and the anatomic adjacency of the aneurysm to the LAD on CTA—collectively supported the diagnosis of external vascular compression by the expanding ventricular aneurysm.

Stenting the compressed segment was deemed high-risk. Therefore, surgical aneurysm resection with CABG was recommended but declined by the patient. He was managed with optimized guideline-directed medical therapy. At one-year follow-up, the patient reported persistent heart failure symptoms (NYHA class II-III). Repeat echocardiography showed the aneurysm was stable in size, but global LV function remained severely impaired (LVEF ∼20%). He had no further hospitalizations for acute heart failure but reported a reduced quality of life. The timeline of the clinical course and management is presented in Table 1.

**Table 1:** Timeline of clinical course and management.

Timepoint	Event	Key findings/interventions
Day 1	Presentation	Chest pain, ECG anterior STEMI, elevated biomarkers.
Day 1–5	Initial Management	Antiplatelet, anticoagulation, heart failure therapy.
Day 5	PCI	Proximal LAD 95% stenosis stented.
Day 7 Discharge		LVEF 43%, on guideline-directed medications.
∼40 days post-discharge	Readmission	Worsening heart failure, LVEF 19%,CAG: new mid-LAD dynamic stenosis.
Readmission period	Advanced Imaging	IVUS, CTA, CMR performed confirming aneurysm compression.
	Treatment Decision	Surgery recommended but declined. Medical therapy continued.
1-year	Follow-up	Aneurysm stable, NYHA II-III, reduced quality of life.

## Discussion

LVA is a common cardiac complication following myocardial infarction. Among patients with acute transmural MI who did not receive reperfusion therapy, approximately 30%–35% develop significant LVA (1). About 85% of these cases occur in the anterior wall or apex, frequently associated with occlusion of the left anterior descending artery—a region where the thinner myocardial structure and relatively poor blood supply increase susceptibility to aneurysmal formation (1). With the widespread use of primary percutaneous coronary intervention, the incidence of LVA has declined markedly in recent years, with current estimates below 5% (2).

LVA represents the end-stage morphological manifestation of post-infarction ventricular remodeling. Pathologically, it results from the replacement of transmural infarcted myocardium by fibrous scar tissue, leading to a persistent, paradoxical bulging of the ventricular wall (1). The remodeling process unfolds in two phases: the early phase, beginning 24–72 h after infarction, involves inflammatory infiltration, cardiomyocyte slippage, and acute expansion of the infarct zone; the late phase, spanning weeks to months, is characterized by fibroblast activation, collagen deposition, and scar maturation. This progression varies considerably among individuals (3). Several factors influence remodeling severity, with anterior wall MI conferring a 1.9-fold higher risk than other infarct locations (4), and multi-vessel disease increasing risk by 1.2-fold compared to single-vessel involvement (5). Systemic inflammation and associated growth factors are central drivers of this process (3); notably, soluble suppression of tumorigenicity 2 (sST2) has been implicated in infarct zone inflammation and is closely linked to myocardial fibrosis and remodeling progression (6).

Giant LVA is now infrequently encountered in clinical imaging, and documented cases of coronary artery compression by an LVA—impairing myocardial perfusion—are rarer still. These uncommon presentations underscore the need to reappraise the clinical implications of LVA. Beyond the conventional risks of thromboembolism, wall motion abnormalities, and heart failure, LVA can initiate a self-perpetuating cycle: myocardial ischemia → ventricular remodeling/LVA formation → coronary compression → recurrent ischemia. This vicious circle significantly elevates the risk of reinfarction, malignant arrhythmias, and sudden cardiac death.

In this particular case of giant LVA, aside from established risk factors such as delayed reperfusion, extensive smoking history, and significant left anterior descending artery disease, the adequacy of pharmacotherapy must be considered. Renin–angiotensin–aldosterone system inhibitors and beta-blockers require careful uptitration to evidence-based target doses, and appropriate diuretic use may mitigate remodeling by reducing ventricular volume load. Additionally, cardiac magnetic resonance revealed extensive microvascular obstruction—a known companion of epicardial reperfusion that may perpetuate ischemic injury and adverse remodeling; thus, strategies to improve microvascular perfusion warrant attention (7). Although this patient declined surgery, LVA resection combined with left ventricular reconstruction remains a well-established curative option for suitable candidates (8).

Emerging molecular targets offer future therapeutic potential. Preclinical studies suggest that inhibiting lysozyme 2 (Lyz2) helps preserve the cardiac extracellular matrix, attenuates scar formation, and improves function (9). Similarly, modulation of the MIAT–DHX9 axis may ameliorate the remodeling microenvironment by regulating abnormal vascular smooth muscle cell activity (10). These approaches target fundamental pathways in fibrosis and inflammation and, though not yet translated to clinical practice, represent promising research directions.

In summary, contemporary understanding frames post-infarction LVA not as a mere passive scar, but as a dynamic, mechanically disruptive entity that actively promotes disease progression. Early recognition and multidisciplinary strategies to disrupt its vicious cycle are essential to improving outcomes, and they reinforce the pathophysiological rationale for surgical intervention in advanced cases.

## Data Availability

The original contributions presented in the study are included in the article/Supplementary Material, further inquiries can be directed to the corresponding author.
